# Community-Acquired Meningitis Complicated by Pyogenic Ventriculitis: A Case Report

**DOI:** 10.7759/cureus.23907

**Published:** 2022-04-07

**Authors:** Pabitra Adhikari, Drashti Antala, Bimatshu Pyakuryal, Ahmed Muhammed, Prasun Pudasainee, Harvey Friedman, Chizoba J Ezepue

**Affiliations:** 1 Internal Medicine, AMITA Health Saint Francis Hospital, Evanston, USA; 2 Critical Care, AMITA Health Saint Francis Hospital, Evanston, USA; 3 Neurology, Sisters of St. Mary (SSM) Health DePaul Hospital, St. Louis, USA

**Keywords:** brudzinski’s sign ventriculitis, brudzinski’s sign, kernig’s sign, intraventricular pus, meningitis, ventriculitis

## Abstract

Ventriculitis is the inflammation of the ependymal lining of the ventricles in the brain which usually occurs as a complication of meningitis, intraventricular devices, intracranial surgery, or brain abscess. Common clinical features include fever, altered mental status, headache, and neck rigidity. Some commonly associated organisms are *Streptococcus,* gram-negative *Bacillus*, *Staphylococcus*, and *Meningococcus.* Here, we report the case of a 57-year-old female presenting with fever, headache, and altered mental status, along with positive physical examination findings of Kernig’s and Brudzinski’s signs without any focal neurological deficits. Cerebrospinal fluid analysis findings were consistent with bacterial infection with neutrophilic leukocytosis, high protein, and low glucose. The blood culture was positive for *Streptococcus pneumoniae*. Magnetic resonance imaging was negative for enhancement of the meninges but showed fluid-filled layering in the ventricles consistent with pyogenic ventriculitis. The patient improved clinically within three days of initiation of empiric antibiotics.

## Introduction

Pyogenic ventriculitis is a rare intracranial infection affecting the ependymal linings. It is characterized by the presence of suppurative fluid in the ventricles and may result from extension of meningitis into the ventricles, rupture of a brain abscess, or following implantation of pathogens after a head injury or neurosurgical procedure. Common clinical features include headache, fever, neck rigidity, and altered mental status. Diagnosis is confirmed by cerebrospinal fluid (CSF) analysis and magnetic resonance imaging (MRI) demonstrating pus in the ventricles, ependymal thickening, or intraventricular loculations. Timely initiation of antibiotics is crucial for better prognosis, and some cases may require additional neurosurgical intervention for drainage of pus, especially if hydrocephalus is developing. Delay in the diagnosis and treatment can lead to hydrocephalus, poor neurologic outcomes, and even death. Here, we present the case of a 57-year-old female presenting with fever, headache, and altered mental status who was diagnosed with pyogenic ventriculitis based on the MRI finding of pus in the ventricles.

## Case presentation

A 57-year-old female with a medical history of intravenous drug abuse presented with headache and left earache which progressed to altered mentation over the course of a day. The patient had a severe holocephalic headache, non-radiating, not relieved by medications, and associated with nausea. She denied photophobia and vomiting, and all other reviews of systems were unremarkable. At baseline, she was alert and oriented to time, place, and person; however, on presentation, she was oriented to self and rapidly declined to being non-verbal and responded only to painful stimuli. There was no history of similar illnesses among the family members.

Physical examination findings were remarkable for a temperature of 101°F at presentation, neck stiffness, positive Kernig’s and Brudzinski’s signs, and no focal neurologic deficits. Other systemic examination findings were unremarkable. Initial lab work (Table 1) revealed a leukocyte count of 37,000/mm^3^ with 90% polymorphonuclear neutrophils (PMNs) and C-reactive protein (CRP) of 16.6 mg/dL. Computed tomography (CT) scan of the head was normal.

**Table 1:** Significant laboratory work (blood) suggestive of infection.

Significant laboratory workup (Blood)	Results	Reference range
White blood cells	37,000 cells/mL	4,000–10,000 cells/mL
Polymorphonuclear neutrophils	90%	40–60%
C-reactive protein	16.6 mg/dL	Less than 10 mg/dL
Blood glucose level	92 mg/dL	60–140 mg/dL

CSF analysis (Table 2) was consistent with bacterial infection including neutrophilic leukocytosis (2,300/mm^3^ of leukocytes with 83% PMNs), high protein (180 mg/dL), and low glucose (<10 mg/dL). Electroencephalogram ruled out epileptic phenomena. CSF culture was negative for organisms; however, urine streptococcal antigens were positive, and blood culture grew *Streptococcus pneumoniae*.

**Table 2:** CSF analysis findings consistent with a bacterial infection. CSF: cerebrospinal fluid

CSF analysis findings	Results	Reference range
White blood cells	2,300 cells/mL	Less than 5 cells/mL
Polymorphonuclear neutrophils)	83%	Less than 2%
Red blood cells	0 cells/mL	0 cells/mL
Sugar	Less than 10 mg/dL	50–80 mg/dL
Protein	180 mg/dL	15–60 mg/dL

**Figure 1:**
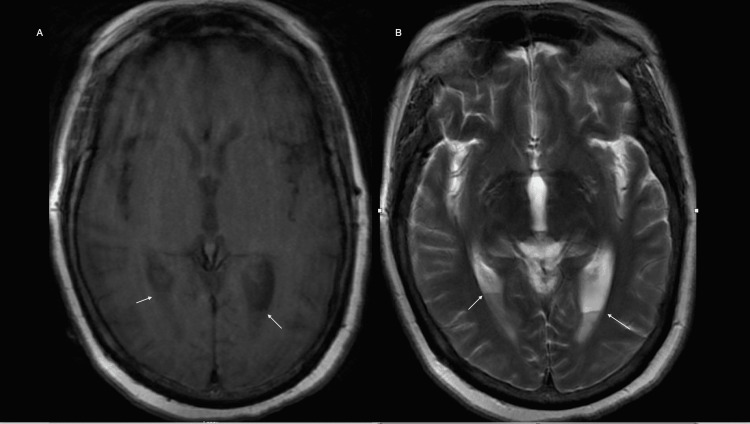
MRI images. (A) Axial T1 image showing the layering of pyogenic material in bilateral occipital horns. (B) Axial T2 image showing the layering of pyogenic material in bilateral occipital horns. MRI: magnetic resonance imaging

MRI (Figures 1A, 1B) demonstrated fluid-fluid level layering in the occipital horns of the lateral ventricle. The patient was started on empiric treatment for meningitis/encephalitis at the time of admission with ceftriaxone, vancomycin, ampicillin, and acyclovir. It was narrowed down to ceftriaxone once *Streptococcus pneumoniae* was isolated from blood. CSF gram stains and cultures were negative likely due to early initiation of antibiotics before lumbar puncture.

The patient improved significantly with the antimicrobial therapy and was back to baseline mental status with the resolution of headache, fever, and neck rigidity, as well as improvement of leukocytosis within three days of treatment. Ceftriaxone was continued for four weeks, and follow-up imaging after completion of antimicrobial therapy revealed resolution of collection in the ventricles.

## Discussion

Pyogenic ventriculitis is an uncommon infection involving the ependymal lining of the ventricles of the brain [1]. It is also known as ependymitis, pyocephalus, or intraventricular empyema [1]. Ventriculitis can develop as a complication of meningitis, neurosurgical procedures, intraventricular drains, or ruptured brain abscesses [1,2]. It can develop as a complication of meningitis if the immunity is compromised due to human immunodeficiency virus, cancer, diabetes, and alcoholism, and the causative organism is highly virulent [2]. Primary community-acquired pyogenic ventriculitis is rare with only a few cases described in the literature [3,4]. There has been no common definition of ventricular infection, and whether to consider it along the ventricular catheter-related infections is unclear [5]. Visualization of ventricular debris on imaging has been a consistent finding pointing to ventriculitis, according to a study done by Fukui et al. [6].

Common organisms associated with ventriculitis include *Streptococcus* (44.9%), gram-negative *Bacillus* (27.6%), and *Staphylococcus *(15.3%) [7]. Oral bacteria such as *Streptococcus pneumoniae*, *Haemophilus influenzae*, and *Streptococcus pyogenes* have been reported in patients with skull base fractures and persistent CSF leaks [2]. Most common initial presentations include fever (92.2%) and altered mental status (82.7%), along with other symptoms such as headache (74.6%), neck stiffness (58.4%), photophobia/phonophobia (40%), and nausea (27.2%) [7]. The triad of fever, altered mental status, and neck stiffness are notable only in one-third of patients [7], which was present in our case. Moreover, focal neurologic deficits (30%) and seizures (18.4%) have been reported, which were not present in our case [7].

CSF analysis findings include elevated protein (greater than 50 mg/dL), low glucose (less than 25 mg/dL), and high leukocytes (more than 10 cells/µL), with neutrophilic predominance (50% or more). CSF cultures may be negative after antibiotic therapy despite active ventriculitis [2], as in our case. Most common abnormalities in brain imaging include the demonstration of intraventricular pus (82.7%), ependymal enhancement (71.4%), and intraventricular loculations (15.3%) [7].

In primary ventriculitis, the ventricles are the major site of infection. Therefore, CSF analysis findings may not always reflect the severity of infection. In case of suspected community-acquired bacterial meningitis, lumbar puncture and starting empirical antibiotics suffice as the initial management. However, in the case of pyogenic ventriculitis, lumbar puncture and empiric antibiotics alone may not be enough. Early detection with MRI brain and neurosurgical intervention, if needed, should go hand in hand. Exclusive dependence on CSF analysis findings may lead to residual infections in the choroid plexus, which can lead to recurrent infection and abscess formation in the future. A duration of 6-12 weeks has been suggested [8], which is similar to the duration used for brain abscesses.

Although neuro-endoscopic surgery has been utilized in patients with ventriculitis caused by shunt infection and malfunction, there are no clear guidelines for its implication for primary pyogenic ventriculitis [9].

Pyogenic ventriculitis can lead to poor neurological function, hydrocephalus, or even death. Timely effective treatment can reduce the possibility of an adverse outcome. A high index of suspicion, early detection with MRI brain, initiation of empiric antibiotics, and early surgical intervention, if indicated, are necessary to prevent poor clinical outcomes. If there are signs and symptoms of persistently elevated intracranial pressure, repeat lumbar puncture or external ventricular drain or shunting may be needed.

Precise guidelines exist for the management of ventricular catheter-related ventriculitis, but there are no strict recommendations nor expert opinions regarding the optimal regimen or duration of treatment for primary bacterial ventriculitis. Treatment with long-term antibiotics and follow-up neurological signs and imaging are the standard of care. There has been a reported case of pneumococcal ventriculitis where clearing up of ventricular pus in MRI was noticed with intravenous ceftriaxone in six weeks [3]. High-quality studies evaluating prognosis are lacking.

In our case, with the initial presentation of fever, left otalgia, acute-onset altered mental status with positive Kernig’s and Brudzinski’s signs on examination, and leukocyte counts of 37,000 mm^3^, meningitis was the top differential diagnosis. Hence, the patient was started on empirical antibiotics for meningitis/encephalitis, including ceftriaxone, vancomycin, ampicillin, and acyclovir to cover viral meningitis/encephalitis. The patient’s MRI demonstrated fluid-fluid level layering in the occipital horns of the symmetrically prominent temporal horns of the lateral ventricle along with left middle ear effusion. The patient’s mental status improved significantly with the antibiotics. With the isolation of *Streptococcus pneumoniae *in blood, antibiotics were narrowed down to ceftriaxone.

Given the left otalgia at presentation and middle ear effusion, it could have been the possible source of meningitis for our patient which could have led to the complication of pyogenic ventriculitis.

## Conclusions

We discuss the case of a patient with community-acquired ventriculitis associated with meningitis who presented with ear pain initially and developed symptoms such as severe headache, followed by meningismus, fever, and altered mental status. Consideration for ventriculitis should be made in the differential, especially among immunocompromised or illicit drug abusers. Timely intervention is necessary in such cases and efforts should be made to obtain a CSF sample before starting antibiotics; however, this should not delay starting treatment for the patient.

Intraventricular debris on MRI can support the diagnosis. There has been no precise guideline for the treatment; however, early initiation of antibiotics, as well as long-term continuation with follow-up neurological examination and imaging are important for better outcomes.
